# Supplementary figures and images for: Mass evacuation and increases in long-term care benefits: Lessons from the Fukushima nuclear disaster
Source: PLoS One. 2019 Sep 25;14(9):e0218835. doi: 10.1371/journal.pone.0218835 (PMC6760790; doi:10.1371/journal.pone.0218835)

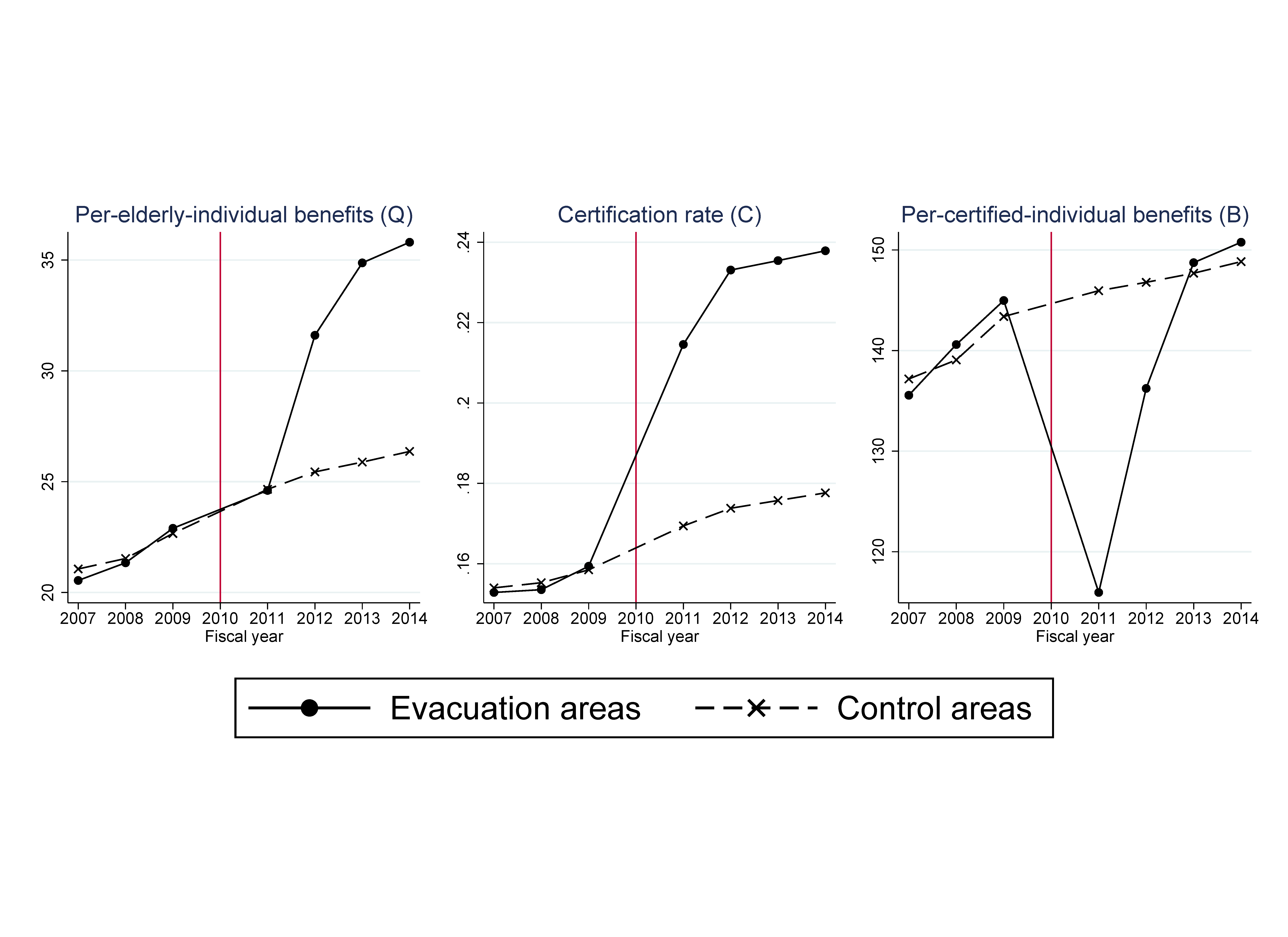

Supplement: S3 Fig — Per-elderly-individual benefits: long-term-care benefits per elderly person. Certification rate: the percentage of people aged 65 and older who were certified to receive long-term care services. Per-certified-individual benefits: long-term care benefits per certified individual. The unit of “benefit” is 1,000 points (10,000 JPY or around 100 USD in standard areas). The trimmed sample includes the municipalities in the baseline sample whose outcome values are within the minimum and maximum of outcome values of the treated municipalities in all three pre-disaster years. (TIFF) [file pone.0218835.s003.tiff]
